# Performance of the WeNMR CS-Rosetta3 web server in CASD-NMR

**DOI:** 10.1007/s10858-015-9942-7

**Published:** 2015-05-17

**Authors:** Gijs van der Schot, Alexandre M. J. J. Bonvin

**Affiliations:** Faculty of Science – Chemistry, Bijvoet Center for Biomolecular Research, Utrecht University, Utrecht, The Netherlands; Laboratory of Molecular Biophysics, Department of Cell and Molecular Biology, Uppsala University, Husargatan 3, Box 596, 75 124 Uppsala, Sweden

**Keywords:** Automated structure determination, Chemical shifts, NOE-based scoring, Grid computing

## Abstract

We present here the performance of the WeNMR CS-Rosetta3 web server in CASD-NMR, the critical assessment of automated structure determination by NMR. The CS-Rosetta server uses only chemical shifts for structure prediction, in combination, when available, with a post-scoring procedure based on unassigned NOE lists (Huang et al. in J Am Chem Soc 127:1665–1674, 2005b, doi:10.1021/ja047109h). We compare the original submissions using a previous version of the server based on Rosetta version 2.6 with recalculated targets using the new R3FP fragment picker for fragment selection and implementing a new annotation of prediction reliability (van der Schot et al. in J Biomol NMR 57:27–35, 2013, doi:10.1007/s10858-013-9762-6), both implemented in the CS-Rosetta3 WeNMR server. In this second round of CASD-NMR, the WeNMR CS-Rosetta server has demonstrated a much better performance than in the first round since only converged targets were submitted. Further, recalculation of all CASD-NMR targets using the new version of the server demonstrates that our new annotation of prediction quality is giving reliable results. Predictions annotated as weak are often found to provide useful models, but only for a fraction of the sequence, and should therefore only be used with caution.

## Introduction

An understanding of the three-dimensional (3D) structure of proteins at atomic resolution and their conformational variability and dynamics, is essential for a proper understanding of their function and their interactions with other proteins and ligands, and for rational drug design (van den Bedem and Fraser 2015). Currently there are several techniques that can produce protein structures at atomic resolution: X-ray crystallography, and nuclear magnetic resonance spectroscopy (NMR), with cryo-electron microscopy (cryo-EM) now reaching atomic resolution with recent advances in detector technology and improved software and algorithms (Bai et al. 2015). NMR is limited in the size of molecules it can study, but has the advantage with respect to other methods that it can study protein dynamics from picosecond up to millisecond time scales and beyond.

The most time-consuming and difficult part of NMR structure elucidation is the assignment of side chain chemical shifts and the NOE cross peaks and several methods have been developed over the years to automate as much as possible this process, often in combination with structure calculations (Guerry and Herrmann 2011). Methods such as CS-ROSETTA (Shen et al. 2008), CHESSHIRE (Cavalli et al. 2007) and CS23D (Wishart et al. 2008) avoid this step by exploiting the structural knowledge present in the readily available backbone chemical shifts. The backbone chemical shifts themselves reflect an appreciable amount of structural information, such as backbone and side-chain conformations, secondary structure, aromatic ring position and the presence of hydrogen bonds. These methods use the backbone chemical shift, together with a database of known protein structures and of their backbone chemical shifts to predict the 3D structure of proteins.

The standard CS-ROSETTA protocol consists of three steps: (1) the selection of fragments; (2) the assembly of models from these fragments; (3) the selection of models. In a recent paper we introduced a number of algorithmic advances for CS-ROSETTA including the rosetta3 fragment picker (R3FP), and a post-analysis procedure that annotates the reliability of predicted structure, and identifies the locally converged regions of the models (van der Schot et al. 2013). These improvements together are shown to improve the reliability, convergence of the final structure. The annotation prediction is based on: (1) the total number of converged residues, (2) the significance of the ROSETTA energy gap, and (3) the quality of the chemical shift data. The label *strong* indicates that the converged regions are likely to be correct, whereas the annotation *weak* indicates that the conserved regions have to be handled with care.

In this work we assess the impact of those recent developments by (re) predicting the structure of 19 CASD-NMR (critical assessment of automated structure determination by NMR) (Rosato et al. 2009, 2012) targets. We used the WeNMR (Wassenaar et al. 2012) webservice CS-ROSETTA3 (https://www.wenmr.eu/wenmr/structure-calculation-software) (van der Schot et al. 2013), connected to the computational resources of the European Grid Initiative (EGI, www.egi.eu), for efficient CS-ROSETTA3 calculations. This service uses the new R3FP fragment picker for fragment selection, distributes the assembly step over the available nodes (using ROSETTA3.3), and implements the new post-analysis procedure (van der Schot et al. 2013). The results are compared to the results from our original structure predictions submitted to CASD-NMR.

## Materials and methods

We evaluated our new structure prediction methodology by predicting the structure of 19 CASD-NMR targets. The targets are named by their respective CASD-NMR and PDB-IDs. They were all provided by the Northeast Structural Genomic Consortium (Huang et al. 2005a), representing a consistent set of data made available via the WeNMR site (https://www.wenmr.eu/wenmr/casd-nmr). We omitted target 2LOJ, due to the large number of unusual and ‘flexible’ amino acids, as we did for the CASD submission. The sequence length of the targets varies between 50 and 149 amino acids, and any flexible termini were excluded from the predictions.

### Fragment selection

The web service CS-ROSETTA3 used R3FP fragment picker for fragment selection. As input only the backbone NMR chemical shift lists were used. Lists can be supplied in any of the NMRPipe(TALOS) (Delaglio et al. 1995), NMR-Star 2.1, or NMR-Star 3.1 (BMRB) formats (Doreleijers et al. 2003).

### Assembly

The web service CS-ROSETTA3 used the selected fragments in the ROSETTA3.3 assembly step. For each target, 50.000 models were generated automatically, using the standard CS-ABRELAX protocol. The model generation step was distributed over the available nodes in the worldwide WeNMR grid under the European Grid Initiative (EGI).

### Conserved regions

The conserved regions of a protein structure prediction were determined using an adaptation of the Gaussian-weighted RMSD method (Damm and Carlson 2006). The 30 lowest ROSETTA energy structures were superimposed using a scaling factor of 2 Å^2^ (Damm and Carlson 2006). This procedure iteratively determines the set of residues on which the structures can be superimposed; residues with a root mean square fluctuation (RMSF) of <2 Å are considered to be converged. Gaps smaller than 3 residues between two low RMSF regions are ignored.

### Annotation

The *cs*-*class*, *convergence* and *energy*-*gap* criteria were used for determining the annotation (van der Schot et al. 2013). The *cs*-*class* criterion is the fraction of residues classified “GOOD” by TALOS+ (Shen et al. 2009). *Convergence* is the fraction of residues, which are considered to be part of a conserved region. The *energy gap* is the difference between the median energy score of the 10 lowest energy score, and the median energy score of the 10 lowest energy models >4 Å away from the best energy model. The gap is directly mapped to [0, 1] using a sigmoidal function. If the predictor model $P_{sum} = 0.08 c_{cs - class} + 0.54  c_{convergence} + 0.38  c_{energy - gap}$ exceeded 0.68, predictions were considered *strong*, and *weak* otherwise (van der Schot et al. 2013).

### Selection of models

The web service uses SPARTA+ (Shen and Bax 2010) to select the final models. For several targets the chemical shift score was combined with the DP score (Huang et al. 2005b). The DP score uses unassigned NOE lists for model selection, which has been shown to improve model selection. Finally the top 5 models after rescoring were used for the comparison step, similarly to the procedure followed for the CASD submissions.

### Evaluation

All Root Mean Square Deviations (RMSDs) are the average RMSD calculated over the C_α_, C, and N atoms, relative to the 20 PDB deposited reference structures, i.e. the average of all pairwise comparisons between the selected models and each of the 20 reference structures in the PDB entry.

## Results

We have compared our original CASD-NMR submissions, both from the first CASD-NMR round, which has been previously evaluated (Rosato et al. 2012) and from the last round, with predictions obtained using the CS-Rosetta3 server (van der Schot et al. 2013), implementing the new R3FP fragment picker for fragment selection. All targets were thus re-run in consistent manner and automatically annotated to evaluate the reliability of the predictions.

### Original CASD-NMR round 2 submissions

Compared to the previous round of CASD-NMR where we submitted prediction irrespective of the convergence of the top 5 models, in this second round we followed a more conservative approach, submitting predictions only for those targets that showed convergence (with as guideline an average RMSD of top 5 models from the best model ~<2 Å). Models were submitted for 7 of the 10 CASD-NMR targets (with HP2876B, StT322 and YR313A unconverged). Convergence and accuracies of these submissions are summarized in Table 1.

**Table 1:** Performance of the CS-Rosetta WeNMR web server in round 2 of CASD-NMR

Target NESG ID^a^	PDB ID	Predicted segment	Server version and scoring^b^	<Pairwise RMSD> of top 5 models^c^	<Pairwise RMSD> from target^c^	<Pairwise RMSD> of target^c^
HR6470A	2L9R	12–58	2.6/CS	0.58 ± 0.18	0.77 ± 0.13	0.47 ± 0.09
HR6430A	2LA6	15–97	2.6/CS–DP	2.28 ± 0.41	2.00 ± 0.30	0.52 ± 0.08
HR5460A	2LAH	13–159	2.6/CS	3.00 ± 0.59	3.38 ± 0.63	0.78 ± 0.12
OR36	2LCI	1–114	2.6/CS–DP	1.13 ± 0.23	1.66 ± 0.27	0.94 ± 0.14
OR135	2LN3	3–76	2.6/CS	0.76 ± 0.27	1.25 ± 0.12	0.74 ± 0.12
HR8254A	2M2E	553–613	2.6/CS	1.46 ± 0.54	1.86 ± 0.34	0.87 ± 0.19
HR2876C	2M5O	16–93	2.6/CS	0.99 ± 0.21	1.33 ± 0.19	0.62 ± 0.09

^a^All targets contributed by the Northeast Structural Genomics Consortium (Huang et al. 2005a) (see Table 2 for references and doi’s)

^b^ *CS* Chemical shift scoring; *DP* DP score (Huang et al. 2005b)

^c^RMSD calculated on backbone CA, C, N atoms

### Prediction and annotation using the CS-Rosetta3 server

Table 2 summarizes the results from the structure predictions for all CASD-NMR targets to date. Six out of nineteen targets were annotated as strong (meaning reliable prediction), and thirteen were annotated weak. Out of the strong targets, on average 86 % of the sequence was regarded as conserved. All strong targets had an average pairwise RMSD within 2 Å from the reference structure, calculated over the conserved regions. One target, 2KPT, converged with the new method (RMSD = 1.39 Å), whereas the original submission did not find the correct fold. For the other strong targets, the results from our new protocol are similar to the performance of the old protocol.

**Table 2:** Performance of the CS-Rosetta WeNMR web server on all recalculated CASD-NMR targets

PDB ID	Target NESG ID^a^		Reference^a^	Rescoring method^b^	Converged regions	Percentage of sequence (%)	Convergence within top5^c^ (RMSD) (Å)	Annotation^d^	RMSD from ref^c^ (Å)	RMSD from ref^c^ (Å) CASD-NMR Submission
2KPT		CGR26A	doi:10.2210/pdb2kpt/pdb	CS	5–44 65–118	79	1.74 ± 0.50	Strong	1.39 ± 0.56	1.41 ± 0.32
2KRU		CtR69A	doi:10.2210/pdb2kru/pdb	CS	1–51	100	0.68 ± 0.08	Strong	0.96 ± 0.14	1.02 ± 0.34
2L9R		HR6470A	doi:10.2210/pdb2l9r/pdb	DP–CS	18–47	64	1.02 ± 0.32	Strong	1.13 ± 0.16	0.77 ± 0.12
2LCI		OR36	doi:10.2210/pdb2lci/pdb	DP–CS	2–44 51–122	90	1.66 ± 0.31	Strong	1.52 ± 0.21	1.43 ± 0.24
2LN3		OR135	Koga et al. (2012)	DP–CS	1–74	100	1.06 ± 0.33	Strong	1.58 ± 0.32	1.00 ± 0.10
2M5O		HR2876C		CS	9–83	85	1.21 ± 0.30	Strong	1.22 ± 0.20	1.15 ± 0.18
2KIF		VpR247	Aramini et al. (2010)	CS	2–33	31	1.32 ± 0.73	Weak	1.10 ± 0.18	1.07 ± 0.23
2KJ6		AR3436A		CS	14–62	55	1.39 ± 0.86	Weak	3.39 ± 0.44	3.75 ± 0.53
2KK1		HR5537A	Liu et al. (2010)	CS	19–68	51	1.22 ± 0.27	Weak	1.21 ± 0.18	1.14 ± 0.07
2KKX		ET109Ared	Wu et al. (2010)	DP–CS	56–77	22	1.58 ± 0.35	Weak	1.38 ± 0.33	0.63 ± 0.04
2KKY		ET109Aox	Wu et al. (2010)	DP–CS	55–77	23	0.91 ± 0.27	Weak	1.16 ± 0.35	0.77 ± 0.03
2KMM		PGR122A	doi:10.2210/pdb2kmm/pdb	CS	32–63	45	0 97 ± 0.22	Weak	1.00 ± 0.09	1.38 ± 0.73
2KNR		AtT13	doi:10.2210/pdb2knr/pdb	CS	75–99	21	1.51 ± 0.63	Weak	2.00 ± 0.17	1.23 ± 0.44
2KPM		NeR103A	doi:10.2210/pdb2kpm/pdb	CS	51–79	35	1.45 ± 0.39	Weak	1.44 ± 0.36	2.17 ± 0.36
2LA6		HR6430A	doi:10.2210/pdb2la6/pdb	DP–CS	60–85	30	0.94 ± 0.16	Weak	0.99 ± 0.13	1.29 ± 0.22
2LAH		HR5460A	doi:10.2210/pdb2lah/pdb	CS	22–50	20	1.19 ± 0.35	Weak	1.05 ± 0.23	1.02 ± 0.27
2LTL		YR313A	doi:10.2210/pdb2ltl/pdb	CS	62–72	10	1.33 ± 0.62	Weak	4.50 ± 0.09	Not Submitted
2LTM		HR2876B	doi:10.2210/pdb2ltm/pdb	CS	63–93	30	1.03 ± 0.28	Weak	0.88 ± 0.20	Not Submitted
2M2E		HR8245A	doi:10.2210/pdb2m2e/pdb	CS	43–67	35	1.32 ± 0.78	Weak	1.48 ± 0.47	0.94 ± 0.13

^a^All targets contributed by the Northeast Structural Genomics Consortium (Huang et al. 2005a)

^b^ *CS* Chemical shift scoring; *DP* DP score (Huang et al. 2005b)

^c^RMSD calculated on backbone CA, C, N atoms of the converged regions

^d^Structure reliability annotation (van der Schot et al. 2013) based on the fraction of residues classified “GOOD” by TALOS+ (Shen et al. 2009), the convergence, defined as the fraction of residues that are considered to be part of a conserved region, and the *energy gap,* the difference between the median energy score of the 10 lowest energy score, and the median energy score of the 10 lowest energy models >4 Å away from the best energy model. See “Materials and methods” for details

For the weak targets, shorter parts of the sequence were regarded as conserved, on average 33 %, with, for 12 out of 13 targets, an average pairwise RMSD from the reference structure 2 Å. The main reason for the weak annotation for those targets is the small fraction of the sequence showing convergence. Our protocol finds the wrong folds for the converged region of target 2KJ6 and 2LTL.

**Fig. 1:**
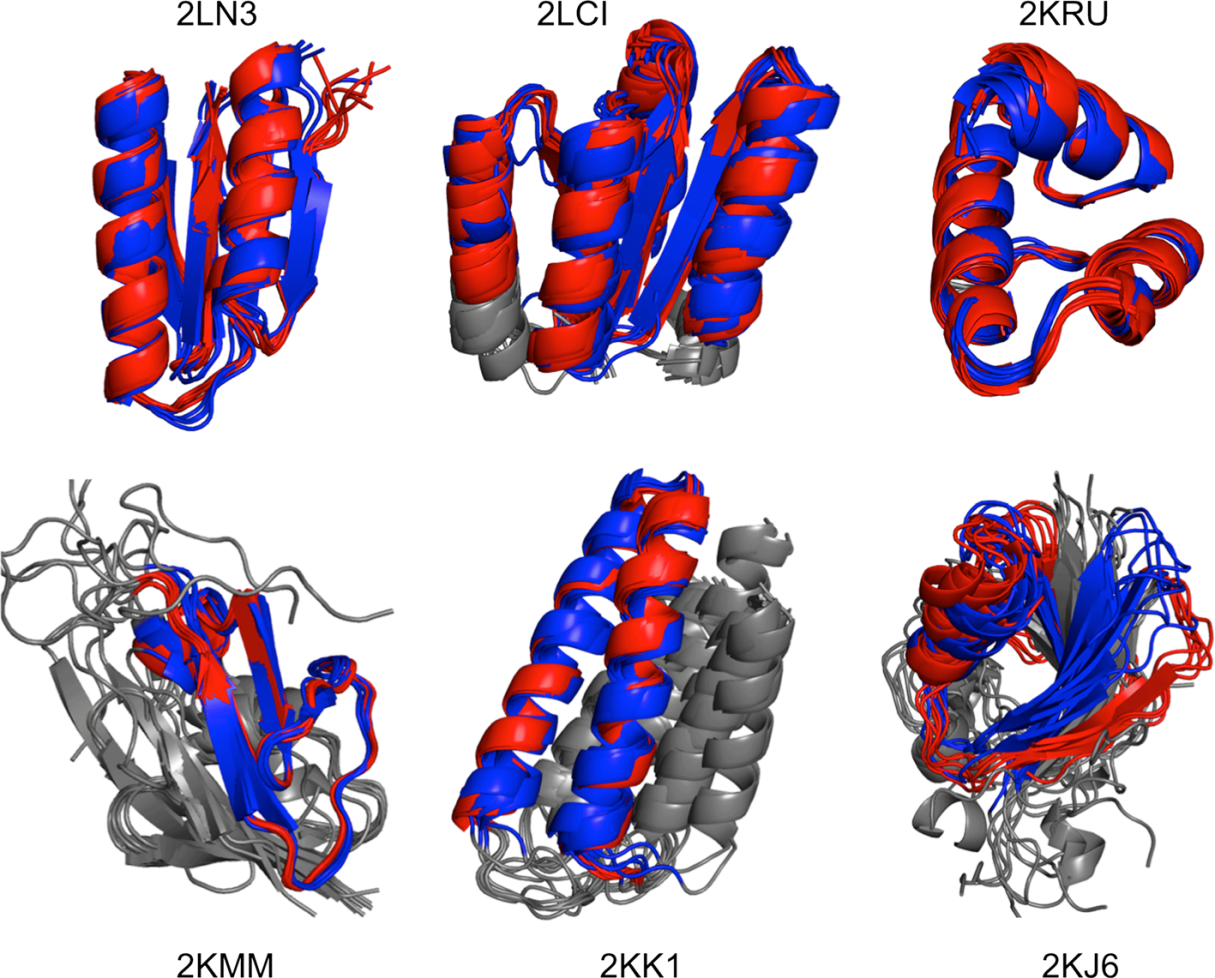
Overview of six representative CASD-NMR targets from the CS-Rosetta3 WeNMR server. The top three structures are annotated as strong (reliable), and the bottom three as weak. For each, the NMR reference structure bundle is in shown in *red*, and the CS-Rosetta3 models in *blue* for the converged regions and *grey* for the unconverged regions

Figure 1 shows an overview of the six strong targets. For each target the reference structures are in blue, and the predicted structures are in red, with unconverged regions in gray.

### Performance of the CS-ROSETTA3 server

**Fig. 2:**
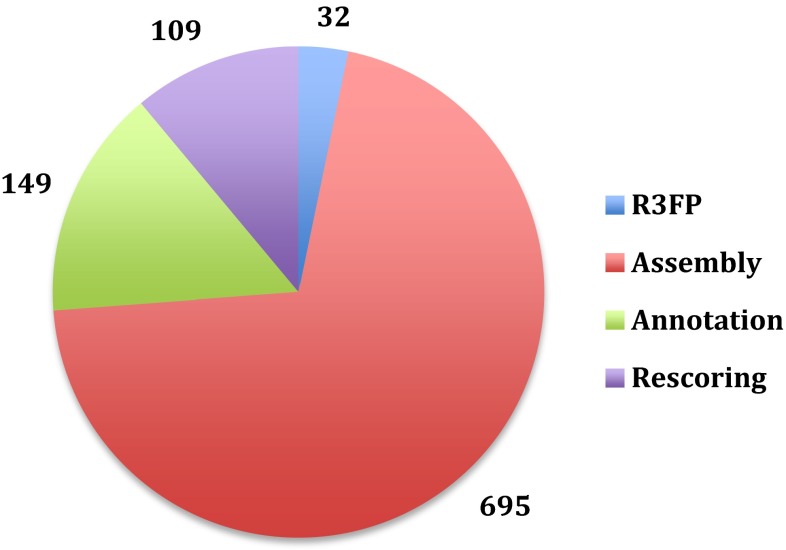
Pie chart showing the CPU time used for an averege CS-Rosetta calculation on the WeNMR grid-enabled server. *Blue* time spent for fragment selection using R3FP (32 min.); *red* assembly time using the WeNMR grid (695 min.); *green* annotation time (149 min.) and *purple* rescoring time (109). An average job takes ~16 h (real time) from submission to completion

Figure 2 shows the average time for each step of the CS-rosetta protocol. On average a complete CS-Rosetta run, including fragment selection, model generation and post-analysis, takes 991 min (16.5 h) on the CS-Rosetta3 WeNMR server. Nearly 45 % of the total time is used to assemble the 50,000 models on the WeNMR EGI grid.

## Discussion

Using the CASD-NMR target, we have shown that, as predicted earlier (van der Schot et al. 2013), our annotation method is able to discriminate successful structure predictions. Six out of 19 targets were annotated as strong. For these targets, the distance from the reference structure was below 2 Å with on average 86 % of the sequence converged. This rather low percentage of strong annotations (31.6 %) leaves space of improvements. For example the RASREC method we have previously published (van der Schot et al. 2013) has been shown to increase the number of strong predictions. This method, however, does require a large number of CPU cores with MPI (Message Passing Interface) communication, which cannot currently be implemented on grid resources.

In the case of weak annotations, the determined “rigid” or converged regions of the predicted model can still be useful: Indeed, in 85 % of those ‘weak’ cases the conserved regions are accurately predicted. However, target 2KJ6 and 2LTL do show that the results of weak predictions have to be used with care. Since 2LTL has only 10 % of its sequence converged, the complete structure should be disregarded, which is an easy case. In contrast, 2KJ6 has 48 % of its sequence converged (a reasonably large fraction), but in fold that is different from the reference structure. Except for the annotation, nothing is really indicative of a wrong fold. We therefore recommend to only use weak annotations with care and search for experimental evidence (e.g. in NOE peaks) of their correctness.

Overall, if we would restrict our earlier submitted models to the conserved regions, we see (Table 2) that we have successfully (RMSD from target <2 Å) predicted the structure for these regions in 88 % of the submitted cases (15 out of 17). Six out of these (40 %) correspond to strong annotations with sequence coverage between 64 and 100 %.

Considering the performance of the grid-enabled web server, we can see that distributing the jobs on the grid speeds-up the calculations ~900 times, compared to running on a single CPU (which would not be a realistic scenario for Rosetta calculations—compared to a 100 CPU cluster the speed up would only be ~9 times). Note that the server is using grid resources in an opportunistic manner, farming out 2500 jobs (for 50,000 models, each jobs calculating 20 models) to grid sites (currently 41 sites are supporting WeNMR (see http://gstat.egi.eu/gstat/geo/openlayers#/VO/enmr.eu) and that grid computations come with some overheads in jobs handling and response.

In conclusion, in this second round of CASD-NMR, the WeNMR CS-Rosetta server has demonstrated a much better performance than in the first round, mainly due to the fact that this time only converged targets were submitted while in the first round all targets were submitted irrespective of their convergence. We have also demonstrated on the recalculated targets that our new annotation of prediction quality is giving reliable results. Our annotations might seem rather conservative considering that more targets annotated as weak show a good similarity to the manual reference structure. These might still provide useful information for further NMR work, but should be used with care.
